# New index using triglyceride glucose-body mass index for predicting mortality in patients with antineutrophil cytoplasmic antibody-associated vasculitis

**DOI:** 10.3389/fmed.2023.1168016

**Published:** 2023-05-19

**Authors:** Pil Gyu Park, Jung Yoon Pyo, Sung Soo Ahn, Jason Jungsik Song, Yong-Beom Park, Ji Hye Huh, Sang-Won Lee

**Affiliations:** ^1^Division of Rheumatology, Department of Internal Medicine, National Health Insurance Service Ilsan Hospital, Goyang, Republic of Korea; ^2^Division of Rheumatology, Department of Internal Medicine, Yonsei University College of Medicine, Seoul, Republic of Korea; ^3^Institute for Immunology and Immunological Diseases, Yonsei University College of Medicine, Seoul, Republic of Korea; ^4^Division of Endocrinology and Metabolism, Department of Internal Medicine, Hallym University Sacred Heart Hospital, Anyang, Republic of Korea

**Keywords:** antineutrophil cytoplasmic antibody-associated vasculitis, mortality, predict, index, TyG-BMI

## Abstract

**Background:**

This study aimed to investigate whether triglyceride glucose-body mass index (TyG-BMI) and a new index using TyG-BMI (NITGB) could predict all-cause mortality in non-obese patients with antineutrophil cytoplasmic antibody (ANCA)-associated vasculitis (AAV).

**Methods:**

The medical records of 78 non-obese AAV patients (BMI < 23.0 kg/m^2^ for Asians) were retrospectively reviewed. TyG-BMI was calculated by the equation: Ln (triglyceride × fasting glucose/2) × BMI. To develop NITGB, we assigned a weight of a number close to an 0.1 decimal integer to each variable according to the slopes for independent variables with *P-*value < 0.1 in the multivariable Cox analysis.

**Results:**

The median age was 54.3 years and five patients died. When non-obese AAV patients were divided into two groups based on TyG-BMI ≥ 187.74, those with TyG-BMI ≥ 187.74 exhibited a significantly higher risk for all-cause mortality than those without (RR 9.450). Since age (HR 1.324), Birmingham vasculitis activity score (BVAS; HR 1.212), and TyG-BMI ≥ 187.74 (HR 12.168) were independently associated with all-cause mortality, NITGB was developed as follows: age + 0.2 × BVAS + 2.5 × TyG-BMI ≥ 187.74. When non-obese AAV patients were divided into two groups based on NITGB ≥ 27.36, those with NITGB ≥ 27.36 showed a significantly higher risk for all-cause mortality than those without (RR 284.000). Both non-obese AAV patients with TyG-BMI ≥ 187.74 and those with NITGB ≥ 27.36 exhibited significantly higher cumulative rates of all-cause mortality than those without.

**Conclusion:**

NITGB along with TyG-BMI could predict all-cause mortality in non-obese AAV patients.

## 1. Introduction

Antineutrophil cytoplasmic antibody (ANCA)-associated vasculitis (AAV) is a cluster of vasculitides affecting mainly the capillaries, arterioles, venules, and occasionally small arteries (1). Its histological features are characterized by typical necrotising vasculitis with few or no immune deposits (1, 2). AAV is generally classified into microscopic polyangiitis (MPA), granulomatosis with polyangiitis (GPA), and eosinophilic granulomatosis with polyangiitis (EGPA) but may be categorized according to antigens targeted by ANCA as follows: MPO-ANCA vasculitis, PR3-ANCA vasculitis, and ANCA-negative vasculitis (3, 4).

A previous study with an inception cohort reported that the overall all-cause mortality rate was 38.4/1,000 patient-years, and the standardized mortality ratio was 2.3. The most common cause of mortality was cardiovascular disease (CVD) with a cumulative incidence rate of 7.1%, followed by malignancy and infection, and the predictor of mortality was the presence of MPO-ANCA (5). Meanwhile, AAV-specific indices for disease activity and prognosis, such as the Birmingham vasculitis activity score (BVAS) and five-factor score (FFS) at AAV diagnosis, have also been reported as independent predictors of CVD or CVD-related mortality in patients with AAV (6, 7). In addition to these indices, the risk factors for all-cause mortality in patients with systemic necrotising vasculitis should be considered (8, 9). We previously reported that both old age and male sex were significantly associated with all-cause mortality in patients with AAV (10, 11). Since the mortality rate of patients with AAV is relatively higher than that of those with other vasculitides (12), more careful attention should be paid to patients with risk factors for all-cause mortality at diagnosis.

Recently, a novel index, triglyceride (TG) glucose-body mass index (TyG-BMI), was introduced. The equation for calculating TyG-BMI consists of three variables including fasting plasma TG level, fasting plasma glucose level, and BMI. As expected from the constituent variables, TyG-BMI was initially proposed as a predictor of type 2 diabetes mellitus (T2DM) and insulin resistance (IR) (13, 14). In addition to T2DM or IR, TyG-BMI was reported to be useful in identifying non-alcoholic fatty liver disease in both the general population with obesity and those without (15, 16), which is closely associated with an increased risk for cardiovascular disease (CVD) (17). Also, TyG-BMI has been demonstrated to be significantly associated with cerebrovascular accidents (CVA; ischaemic stroke) in the general population (18).

Given that IR and its related diseases such as CVD and CVA are generally major risk factors for all-cause mortality in the general population, it could be assumed that TyG-BMI could be a robust predictor of all-cause mortality in AAV patients. However, to date, no study has evaluated the predictive potential of TyG-BMI for unwanted outcomes such as all-cause mortality, CVA, and CVD in AAV patients. Hence, we included only non-obese AAV patients (BMI < 23.0 kg/m^2^ for Asians) in this study to minimize the effect of BMI on mortality (19, 20) and investigated whether TyG-BMI at AAV diagnosis could predict poor outcomes during follow-up in non-obese AAV patients. In addition, when TyG-BMI and other variables were competitive in predicting all-cause mortality, we attempted to develop a new index for predicting all-cause mortality based on TyG-BMI and evaluated its clinical usefulness in non-obese AAV patients.

## 2. Methods

### 2.1. Patients

A total of 141 AAV patients who fulfilled the inclusion criteria and did not meet the exclusion criteria were selected from the Severance Hospital ANCA associated VasculitidEs (SHAVE) cohort. Among them, 78 patients were non-obese. The inclusion criteria have been previously described elsewhere (21, 22): (1) initial diagnosis of AAV at the Division of Rheumatology, the Department of Internal Medicine, Yonsei University College of Medicine, Severance Hospital, from October 2000 to May 2021; (2) fulfillment of the 2012 revised International Chapel Hill Consensus Conference Nomenclature of Vasculitides and the 2007 European Medicine Agency algorithm for AAV; (3) well-documented medical records sufficient to collect clinical and laboratory data including ANCA results at AAV diagnosis, calculate BVAS and FFS at AAV diagnosis, and analyse unwanted outcomes during follow-up (6, 7); (4) available data on the variables that compose the equation to calculate TyG-BMI such as fasting plasma TG level, fasting plasma glucose level, and BMI at AAV diagnosis; and (5) BMI < 23.0 kg/m^2^. According to the World Health Organization BMI classification for the Asian population, non-obese patients were defined as those with BMI < 23.0 kg/m^2^ (19). Of the 141 patients, the final 78 non-obese AAV patients were included in this study and their medical records were retrospectively reviewed. To minimize ethnic differences, only Korean patients with AAV were included in this study. The exclusion criteria have been previously described elsewhere (21, 22): (1) concurrent serious medical conditions such as cancers, infectious diseases that require hospitalization, and other systemic vasculitides at AAV diagnosis; (2) follow-up duration of < 3 months after the time of AAV diagnosis; and (3) exposure to immunosuppressive drugs for treating suspected AAV in outside hospitals before AAV diagnosis. The present study was approved by the Institutional Review Board (IRB) of Severance Hospital (Seoul, Republic of Korea, IRB No. 4-2020-1071), and conducted in accordance with the Declaration of Helsinki. Given the retrospective design of the study and the use of anonymised patient data, the requirement for written informed consent was waived.

### 2.2. Variables

The collected variables are described in Table 1. Patient baseline data, including basic patient characteristics, physical examination findings (e.g., BMI), and laboratory data, were collected by trained research coordinators at each visit. Blood samples for biochemical analysis were obtained after an overnight fasting period of at least 8 h. All-cause mortality was defined as death from any etiology. The follow-up duration based on all-cause mortality was defined as the period between AAV diagnosis and the last visit for surviving patients and as the period between AAV diagnosis and death for deceased patients. Medications were defined as those that had been administered from AAV diagnosis to the last visit.

**Table 1:** Characteristics of non-obese AAV patients (BMI < 23.0 kg/m^2^).

**Variables**	**Values**
**At diagnosis**	
**Basic clinical data**	
Age (years)	54.3 (27.8)
Male sex [*N*, (%)]	16 (20.5)
**AAV subtypes [** ***N*** **, (%)]**	
MPA	42 (53.8)
GPA	16 (20.5)
EGPA	20 (25.6)
**ANCA positivity [** ***N*** **, (%)]**	
MPO-ANCA (or P-ANCA) positive	50 (64.1)
PR3-ANCA (or C-ANCA) positive	17 (21.8)
**AAV-specific indices**	
BVAS	13.0 (12.0)
FFS	1.0 (2.0)
**Comorbidities [** ***N*** **, (%)]**	
T2DM	14 (17.9)
Hypertension	17 (21.8)
**Acute phase reactants**	
ESR (mm/h)	48.0 (60.0)
CRP (mg/L)	3.6 (27.6)
**TyG-BMI index-related variables**	
TyG	8.6 (0.7)
BMI (kg/m^2^)	19.9 (3.0)
TyG-BMI	171.1 (26.6)
**During follow-up**	
**Poor prognosis [** ***N*** **, (%)]**	
All-cause mortality	5 (6.4)
Follow-up period based on all-cause mortality	36.6 (68.6)
**Medications [** ***N*** **, (%)]**	
Glucocorticoids	74 (94.9)
Cumulative dose of glucocorticoids (mg, equivalent to prednisolone)	9,597.5 (10,466.0)
Cyclophosphamide	39 (50.0)
Rituximab	9 (11.5)
Mycophenolate mofetil	15 (19.2)
Azathioprine	41 (52.9)
Tacrolimus	8 (10.3)
Methotrexate	7 (9.0)
Plasma exchange	6 (7.7)

Values are expressed as a median (interquartile range, IQR) or N (%).

AAV, ANCA-associated vasculitis; ANCA, antineutrophil cytoplasmic antibody; BMI, body mass index; MPA, microscopic polyangiitis; GPA, granulomatosis with polyangiitis; EGPA, eosinophilic granulomatosis with polyangiitis; MPO, myeloperoxidase; P, perinuclear; PR3, proteinase 3; C, cytoplasmic; BVAS, Birmingham vasculitis activity score; FFS, five-factor score; T2DM, type 2 diabetes mellitus; ESR, erythrocyte sedimentation rate; CRP, C-reactive protein; TyG, triglyceride glucose; TyG-BMI, triglyceride glucose-body mass index.

### 2.3. Equations of TyG-BMI and a new index using TyG-BMI for all-cause mortality

TyG-BMI is calculated as follows: Ln [TG (mg/dL) × FPG (mg/dL)/2] × BMI (14, 23). To develop an equation of a new index using TyG-BMI and other variables for predicting all-cause mortality, we assigned a weight of a number close to an 0.1 decimal integer to each variable according to the slopes for independent variables with *P-*value < 0.1 in the multivariable Cox analysis as described in our previous study (24).

### 2.4. Statistical analysis

All statistical analyses were performed using IBM SPSS Statistics for Windows, version 26 (IBM Corp., Armonk, NY, USA). Continuous variables were expressed as medians (interquartile ranges) and categorical variables as numbers (percentages). The correlation coefficient (r) between the two variables was obtained using Pearson correlation analysis. The area under the curve (AUC) was calculated using the receiver operating characteristic (ROC) curve analysis. Furthermore, the optimal cutoff value was extrapolated by performing the ROC curve analysis, and one value with the maximum sum of sensitivity and specificity was selected. The multivariable Cox hazard model using variables with statistical significance in the univariable Cox hazard model was used to obtain the hazard ratios (HRs) during follow-up. The relative risk (RR) of the cutoff value for high AAV activity was analyzed using contingency tables and the chi-square test. The cumulative survival rates between the two groups were compared using the Kaplan–Meier survival analysis with the log-rank test. Statistical significance was set at *P* < 0.05 in the overall analyses and *P* < 0.1 in the ROC curve and univariable and multivariable Cox analyses.

## 3. Results

### 3.1. Patients characteristics

At AAV diagnosis, the median age was 54.3 years and 20.5% of the patients were men. In total, 42, 16, and 20 patients were classified as having MPA, GPA, and EGPA, respectively. MPO-ANCA (or P-ANCA) and PR3-ANCA (or C-ANCA) were positive in 50 (64.1%) and 17 patients (21.8%), respectively; 14 patients had T2DM and 17 had hypertension. The median BVAS, FFS, erythrocyte sedimentation rate (ESR), and C-reactive protein (CRP) level were 13.0, 1.0, 48.0 mm/h, and 3.6 mg/L, respectively. Also, the median TyG, BMI, and TyG-BMI were 8.6, 19.9 kg/m^2^, and 171.1, respectively. During follow-up, five patients (6.4%) died within an average follow-up duration of 36.6 months. In addition, six patients (7.7%) had CVA and four patients (5.1%) had CVD but no statistical significance was found. Glucocorticoids, cyclophosphamide, and azathioprine were administered to 74 (94.9%), 39 (50.0%), and 41 patients (52.9%), respectively. The median cumulative dose of glucocorticoids, which was equivalent to prednisolone, was 9,597.5 mg (Table 1), and those administered to surviving and deceased patients were 9,631.0 mg and 8,120.0 mg, respectively.

### 3.2. Correlations of TyG-BMI with the continuous variables at AAV diagnosis

TyG-BMI was not significantly correlated with age (*r* = 0.168), BVAS (*r* = −0.184), FFS (*r* = *-*0.071), ESR (*r* = 0.138), and CRP level (*r* = *-*0.073).

### 3.3. Comparison of the AUCs of TyG-BMI for predicting all-cause mortality according to BMI

**Figure 1:**
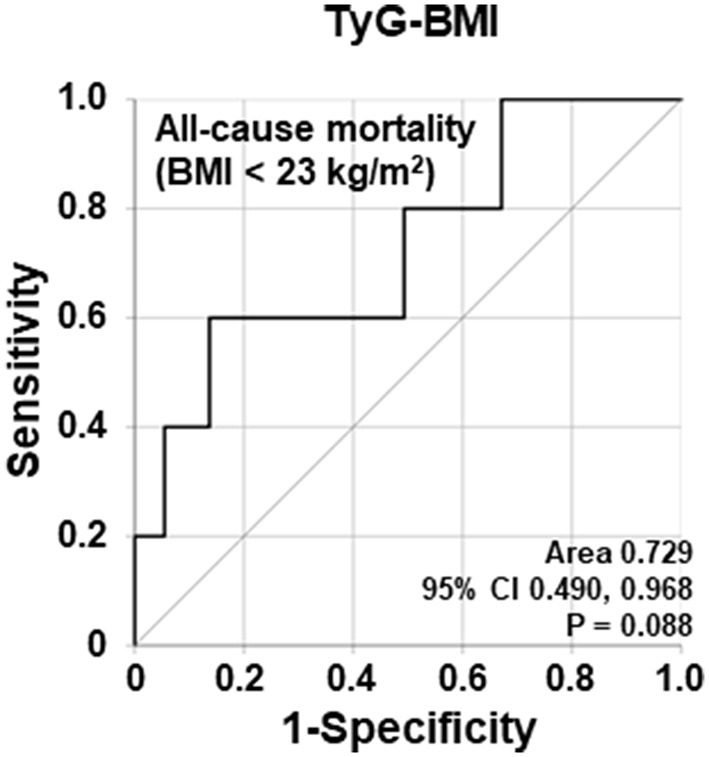
Comparison of area under the curves of TyG-BMI for all-cause mortality based on BMI < 23.0 kg/m^2^. The AUC of TyG-BMI for all-cause mortality in non-obese AAV patients tended to be statistically significant. TyG, triglyceride glucose; BMI, body mass index; AAV, ANCA-associated vasculitis; ANCA, antineutrophil cytoplasmic antibody.

Using the ROC curve, the AUC of TyG-BMI in the 78 non-obese AAV patients was significant (AUC = 0.729, *P* = 0.088) (Figure 1).

### 3.4. Comparison of the AUCs of TyG-BMI for predicting other poor outcomes

Poor outcomes other than all-cause mortality, including relapse, end-stage renal disease (ESRD), CVA, and CVD, were also analyzed. The follow-up duration based on each poor outcome was defined as the period between AAV and each poor outcome occurrence. Conversely, for patients who had no poor outcome, it was defined as the period between AAV diagnosis and the last visit. In this study, the AUCs of TyG-BMI for relapse, ESRD, CVA, and CVD showed no statistical significance in non-obese AAV patients (Supplementary Figure 1).

### 3.5. Optimal cutoff value of TyG-BMI and RR for predicting all-cause mortality

**Figure 2:**
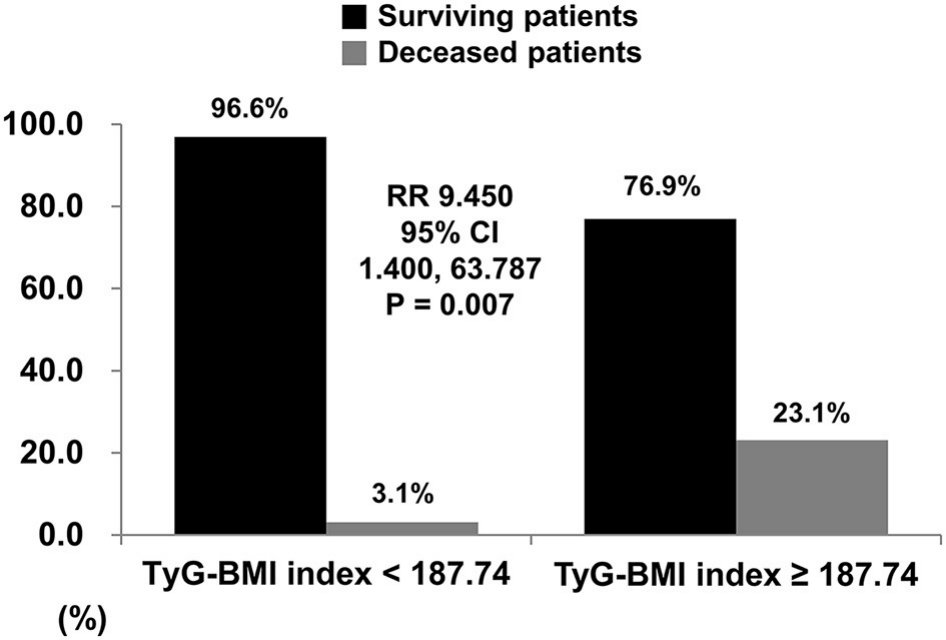
Relative risk of TyG-BMI for all-cause mortality. When the cutoff of TyG-BMI was set as 187.74 using the ROC curve, all-cause mortality was identified in non-obese AAV patients with TyG-BMI ≥ 187.74 more frequently than those with TyG-BMI < 187.74 (23.1% vs. 3.1%, relative risk 9.450, *P* = 0.007). TyG, triglyceride glucose; BMI, body mass index; ROC, receiver operator characteristic; AAV, ANCA-associated vasculitis; ANCA, antineutrophil cytoplasmic antibody.

When the cutoff value of TyG-BMI for all-cause mortality was set as 187.74 in the ROC curve analysis, the sensitivity and specificity were 60.0 and 86.3%, respectively. When non-obese AAV patients were divided into two groups based on this cutoff value, 13 patients were assigned as a group with TyG-BMI ≥ 187.74. All-cause mortality was identified in non-obese AAV patients with TyG-BMI ≥ 187.74 more frequently than those with TyG-BMI < 187.74 (23.1 vs. 3.1%, *P* = 0.007). Furthermore, non-obese AAV patients with TyG-BMI ≥ 187.74 exhibited a significantly higher risk for all-cause mortality than those with TyG-BMI < 187.74 (RR 9.450, 95% confidence interval [CI] 1.400, 63.787) (Figures 1, 2).

### 3.6. Cox hazards analyses based on TyG-BMI ≥ 187.74

In the univariable analysis, age (HR 1.359), male sex (HR 7.656), and TyG-BMI ≥ 187.74 (HR 6.264) were significantly associated with all-cause mortality, and BVAS (HR 1.116) tended to be associated with all-cause mortality in non-obese AAV patients (*P* = 0.060). As described in the Methods section, variables with a *P-*value < 0.1 were included in the multivariable Cox analysis. In the multivariable analysis, none of the variables were statistically significant. However, age (HR 1.324, *P* = 0.089), BVAS (1.212, *P* = 0.095), and TyG-BMI ≥ 187.74 (HR 12.168, *P* = 0.056) tended to be associated with all-cause mortality meaningfully in non-obese AAV patients (Table 2).

**Table 2:** Cox hazards model analysis of variables at diagnosis for all-cause mortality during follow-up in non-obese AAV patients (BMI < 23.0 kg/m^2^).

**Variables**	**Univariable**			**Multivariable**			
	**HR**	**95% CI**	***P*** **-value**	**B (slope)**	**HR**	**95% CI**	***P-*** **value**
Age	1.359	1.041, 1.773	0.024	0.281	1.324	0.958, 1.829	0.089
Male sex	7.656	1.239, 46.191	0.026	0.685	1.984	0.234, 16.793	0.530
MPA	58.987	0.044, 79,750.522	0.268				
GPA	0.035	0.000, 397.710	0.481				
MPO-ANCA (or P-ANCA) positive	3.504	0.345, 35.604	0.289				
PR3-ANCA (or C-ANCA) positive	0.034	0.000, 335.249	0.471				
BVAS	1.116	0.995, 1.250	0.060	0.193	1.212	0.967, 1.520	0.095
FFS	1.851	0.803, 4.268	0.149				
T2DM	3.063	0.507, 18.499	0.222				
Hypertension	1.923	0.315, 11.723	0.478				
ESR	1.005	0.982, 1.028	0.661				
CRP	1.002	0.984, 1.020	0.832				
TyG-BMI index ≥ 187.74	6.264	1.038, 37.785	0.045	2.499	12.168	0.937, 157.965	0.056

AAV, ANCA-associated vasculitis; ANCA, antineutrophil cytoplasmic antibody; BMI, body mass index; MPA, microscopic polyangiitis; GPA, granulomatosis with polyangiitis; MPO, myeloperoxidase; P, perinuclear; PR3, proteinase 3; C, cytoplasmic; BVAS, Birmingham vasculitis activity score; FFS, five-factor score; T2DM, type 2 diabetes mellitus; ESR, erythrocyte sedimentation rate; CRP, C-reactive protein; TyG-BMI, triglyceride glucose-body mass index.

### 3.7. New index using TyG-BMI for all-cause mortality

According to the description in the Methods section, three variables, age, BVAS, and TyG-BMI ≥ 187.74, were included in an equation of NITGB for all-cause mortality, their slopes were modified as 0.3 (0.281), 0.2 (0.193), and 2.5 (2.499), and, eventually, an equation was developed as follows: NITGB for all-cause mortality in non-obese AAV patients at AAV diagnosis = 0.3 × age + 0.2 × BVAS + 2.5 × TyG-BMI ≥ 187.74 (yes = 1 and no = 0).

### 3.8. Optimal cutoff value of NITGB and RR for predicting all-cause mortality

**Figure 3:**
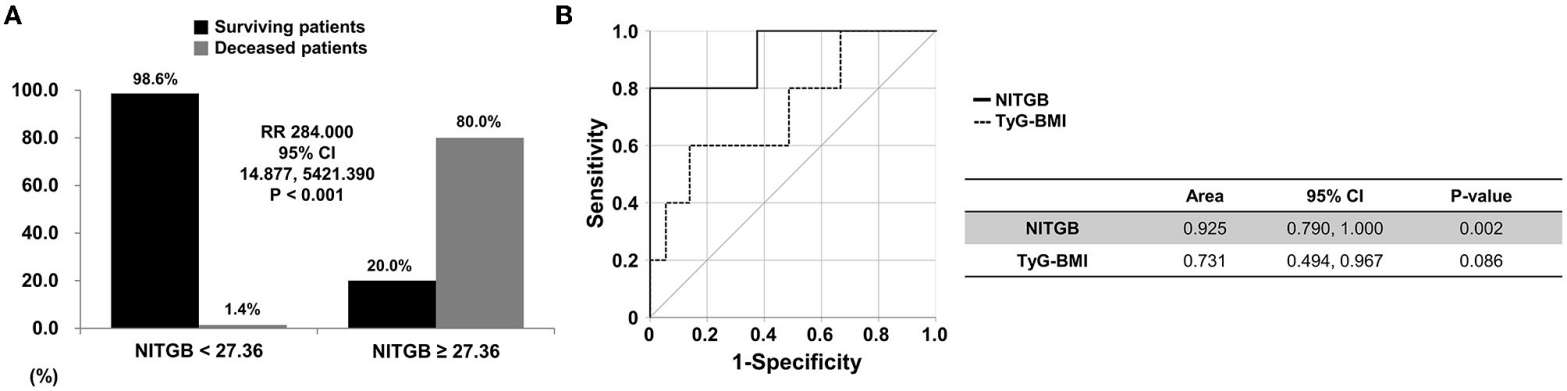
Relative risk of NITGB for all-cause mortality and comparison between TyG-BMI and NITGB. **(A)** When the cutoff value of NITGB was set as 27.36 using the ROC curve, all-cause mortality was found in non-obese AAV patients with NITGB ≥ 27.36 more frequently than those with NITGB < 27.36 (80.0 vs. 1.4%, relative risk 284.000, *P* < 0.001); **(B)** The AUC of NITGB (line) was significantly higher than that of TyG-BMI (dotted) for predicting all-cause mortality in non-obese AAV patients. NITGB, new index using TyG-BMI; TyG, triglyceride glucose; BMI, body mass index; ROC, receiver operator characteristic; AAV, ANCA-associated vasculitis; ANCA, antineutrophil cytoplasmic antibody.

Using the ROC curve, when the cutoff value of NITGB was set as 27.36, the sensitivity and specificity were 80.0 and 98.6%, respectively. When non-obese AAV patients were divided into two groups based on the cutoff value of 27.36, all-cause mortality was observed in non-obese AAV patients with NITGB ≥ 27.36 more frequently than those with NITGB < 27.36 (80.0 vs. 1.4%, *P* < 0.001). Furthermore, non-obese AAV patients with NITGB ≥ 27.36 showed a significantly higher risk for all-cause mortality than those with NITGB < 27.36 (RR 284.000, 95% CI 14.877, 5421.390) (Figure 3A).

### 3.9. Comparison of the AUCs between TyG-BMI and NITGB for all-cause mortality

The AUC of NITGB (area 0.925, *P* = 0.002) was significantly higher than that of TyG-BMI (area 0.731, *P* = 0.088) for predicting all-cause mortality in non-obese AAV patients (Figure 3B).

### 3.10. Comparison of the cumulative rates of all-cause mortality

**Figure 4:**
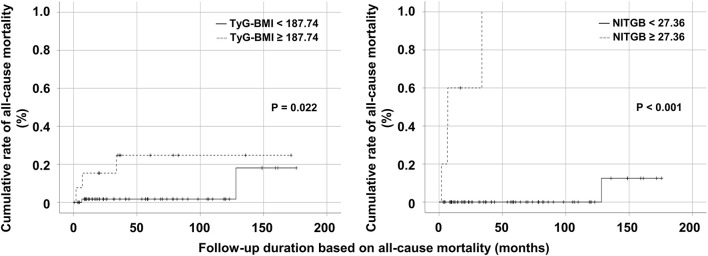
Comparison of the cumulative rates of all-cause mortality. Both non-obese AAV patients with TyG-BMI ≥ 187.74 and those with NITGB ≥ 27.36 exhibited higher cumulative rates of all-cause mortality than those with TyG-BMI < 187.74 and those with NITGB < 27.36. AAV, ANCA-associated vasculitis; ANCA, antineutrophil cytoplasmic antibody; TyG, triglyceride glucose; BMI, body mass index; NITGB, new index using TyG-BMI.

In the comparison based on the cutoff value of TyG-BMI, non-obese AAV patients with TyG-BMI ≥ 187.74 exhibited a significantly higher cumulative rate of all-cause mortality than those with TyG-BMI < 187.74 (*P* = 0.022). In the comparison according to the cutoff value of NITGB, the cumulative rate of all-cause mortality in non-obese AAV patients with NITGB ≥ 27.36 was significantly higher than that in those with NITGB < 27.36 (*P* < 0.001) (Figure 4). Both the cutoff values showed a possibility of predicting all-cause mortality in non-obese AAV patients properly.

## 4. Discussion

Herein, we investigated the ability of TyG-BMI to predict all-cause mortality, developed a new index for predicting mortality using TyG-BMI, and evaluated its clinical usefulness in non-obese AAV patients. We obtained several interesting findings. First, the AUC of TyG-BMI for all-cause mortality in the 78 non-obese AAV patients tended to be statistically significant but those of all obese AAV patients showed no similar trend of significance. Second, when the cutoff value of TyG-BMI for all-cause mortality was set as 187.74, all-cause mortality was more frequently identified in AAV patients with TyG-BMI ≥ 187.74 than those with TyG-BMI < 187.74. Third, a new index using TyG-BMI for all-cause mortality (NITGB) was developed using variables that were with a *P-*value of < 0.1 in the Cox hazards model analyses for predicting all-cause mortality. Fourth, when the cutoff value of NITGB for predicting all-cause mortality was set as 27.36, the cumulative mortality rate in non-obese AAV patients with NITGB ≥ 27.36 was significantly higher than that in those with NITGB < 27.36. Therefore, it is concluded that TyG-BMI and NITGB at AAV diagnosis could be useful in predicting all-cause mortality, and the predictive ability of NITGB is greater than that of TyG-BMI in non-obese AAV patients.

Only non-obese AAV patients were included herein. BMI is known to be closely associated with mortality in the general population. In particular, it shows a U shape, which is characterized by roughly opposite results between the non-obese and obese people (20). Since BMI is proportional to the probability of all-cause mortality in individuals with BMI ≥ 23.0 kg/m^2^, the mortality-reflected effect of BMI itself is added to the predictability of all-cause mortality. TyG may interfere with interpreting the predictability of all-cause mortality of TyG-BMI and leave its lower reliability. Whereas, since BMI is inversely correlated with the probability of all-cause mortality in individuals with BMI < 23.0 kg/m^2^, it may highlight the predictability of all-cause mortality of TyG-BMI in AAV patients. Actually, when all patients were analyzed in this study, the AUC of TyG-BMI for all-cause mortality was not significant, whereas it showed a tendency to be statistically significant in 78 non-obese AAV patients in the ROC curve analysis. These results indicated that the risk of all-cause mortality associated with TyG-BMI is meaningful in non-obese people but it is not in overweight and obese people (Figure 1). This finding is consistent with the findings of other studies which showed that non-obese people have a higher risk of TyG-BMI-related diabetes and non-alcoholic fatty liver disease than overweight and obese people (13, 15). However, we were not able to provide evidence regarding whether the presence or absence of a U shape exist in the association between the risk of all-cause mortality and BMI in our data because the number of mortality case was small.

Since TyG-BMI consists of fasting plasma TG, fasting plasma glucose, TyG, and BMI, we assessed the abilities of each component for predicting all-cause mortality and compared them with TyG-BMI in non-obese AAV patients. First, in the ROC curve analysis, the AUCs of fasting plasma TG, fasting plasma glucose, TyG, and BMI were 0.547 (*P* = 729), 0.521 (*P* = 0.878), 0.570 (*P* = 0.603), and 0.688 (*P* = 0.162), respectively. There was no trend of the significant association between each component composing an equation of TyG-BMI and all-cause mortality (Supplementary Figure 2). Next, in the univariable Cox hazards model analysis, fasting plasma TG (HR 1.001, *P* = 0.908), fasting plasma glucose (HR 1.010, *P* = 0.509), TyG (HR 1.831, *P* = 0.536), and BMI (HR 1.541, *P* = 0.186) were not associated with all-cause mortality. Whereas TyG-BMI ≥ 187.74 (HR 6.264, *P* = 0.045) was independently and significantly associated with all-cause mortality in non-obese AAV patients. Therefore, to cope with the possibility of all-cause mortality during follow-up in non-obese AAV patients, attention should be carefully paid to the predictability of TyG-BMI over the cutoff for predicting mortality at the time of AAV diagnosis.

In this study, to meet the clinical need for a new index for predicting all-cause mortality, the cutoff value of TyG-BMI with a *P-*value of < 0.1 was obtained from the ROC curve. To overcome this statistical limitation, we also analyzed the implication of TyG-BMI for presupposing all-cause mortality using two more cutoffs, the highest tertile and quartile, that are frequently and clinically applied. In terms of the highest tertile of TyG-BMI, the lower limit of the highest tertile was set as 179.28. When non-obese AAV patients were partitioned into two groups, there was no significant difference in the cumulative mortality rates between patients with TyG-BMI ≥ 179.28 and those with TyG-BMI < 179.28 (*P* = 0.276) (Supplementary Figure 3A). In terms of the highest quartile of TyG-BMI, the lowest value of the highest quartile was calculated as 183.57. When non-obese AAV patients were divided into two groups, patients with TyG-BMI ≥ 183.57 tended to exhibit a significantly higher cumulative mortality rate than those with TyG-BMI < 183.57, but it did not reach statistical significance (Supplementary Figure 3B). Therefore, it is concluded that 187.74 obtained from the ROC curve is the suitable cutoff value for predicting all-cause mortality in non-obese AAV patients.

TyG is an index composed of fasting plasma TG and fasting plasma glucose and has been well-known as an index that effectively reflects IR and overall metabolic status in individuals (25). However, Er et al. found that the new index of TyG-BMI, which is formed by combining the TyG index with BMI, can better reflect IR status than the TyG index (13, 14). Our study results provide clinically supporting evidence for the biologically plausible hypothesis that IR plays an important role in AAV patients. Although the underlying mechanism of the relationship between TyG-BMI and all-cause mortality in AAV patients is unclear, it may be related to IR. TyG as a surrogate marker of IR is associated with metabolic disorders including T2DM. Consequently, it ultimately increases the risk of all-cause mortality by enhancing the possibility of systemic complications including CVA and CVD (26). However, when the association between TyG-BMI and CVA or ACS was investigated using the ROC curve and univariable Cox hazards model analysis, the AUCs of TyG-BMI for CVA and CVD were not statistically significant (Supplementary Figure 1), and TyG-BMI was significantly associated with neither CVA (HR 1.026, *P* = 0.315) nor CVD (HR 1.033, *P* = 0.293).

In Table 2, in the multivariable Cox analysis, in addition to TyG-BMI ≥ 187.74, both age and BVAS were also associated with all-cause mortality in non-obese AAV patients. First of all, variables of age and the male sex are ready-established conventional risk factors for all-cause mortality (8). In the previous studies, we demonstrated that elderly AAV patients, as well as male AAV patients, had a significantly higher rate of all-cause mortality (10, 11). However, a variable of age could predict all-cause mortality independently but that of the male sex could not in this study. It is assumed that these results might be owing to a relatively small proportion of male patients compared to females. Furthermore, the inclusion of only AAV patients with BMI < 23.0 kg/m^2^ might have influenced the results by diminishing the effect of obesity on all-cause mortality. On the other hand, BVAS is the most widely used index for assessing the activity of AAV (6). In addition to a role to reflect the cross-sectional activity of AAV, BVAS has been considered associated with all-cause mortality, in particular, cardiovascular disease-related mortality in AAV patients (27, 28).

Given these concepts, in this study, we assigned weights to age, BVAS, and TyG-BMI ≥ 187.74 by referring to the slope of the multivariable Cox analysis, and developed a new index using TyG-BMI for predicting all-cause mortality in non-obese AAV patients. We found two advantages of NITGM compared to TyG-BMI. One is that NITGM exhibited a more robust ability for predicting all-cause mortality than TyG-BMI itself because the abilities of age and BVAS for predicting all-cause mortality were added to that of TyG-BMI. The other is that NITGM includes three risk factors for all-cause mortality in a variety of areas: metabolic disorders-related cardiovascular (TyG-BMI), conventional (age), and AAV-specific (BVAS) risk factors. Finally, we found that the independent ability of NITGM ≥ 27.36 to predict all-cause mortality tended to be stronger than that of TyG-BMI ≥ 187.74 by the comparative analysis of the cumulative rates of all-cause mortality between the two groups (Figure 4).

On the other hand, the cumulative dose or daily dose of glucocorticoids can reasonably be expected to influence the results of this study by affecting the variable of TyG-BMI as well as acting as a risk factor for all-cause mortality. Glucocorticoids may affect glucose metabolism by increasing fasting glucose and insulin resistance and may enhance the frequency of metabolic syndrome occurrence. Therefore, we compared the total amount of glucocorticoids administered during follow-up between surviving and deceased patients but we could find no significant difference between the two groups. Since it was not statistically significant, we did not consider the cumulative dose of glucocorticoids as an independent predictor of all-cause mortality in non-obese AAV patients in this study.

For the first time, we demonstrated the abilities of TyG-BMI and a new index using TyG-BMI (NITGB) to predict all-cause mortality in non-obese AAV patients. Our findings provide evidence that these indices are reliable markers for the early identification of individuals at high risk of mortality. Given the relatively high mortality rate of AAV, it is believed that this study has an advantage in that it enabled the development of biomarkers or indices at the time of AAV diagnosis for predicting all-cause mortality during follow-up in non-obese AAV patients. Additionally, the evaluated indices can be flexibly applied to AAV patients with ethnic and geographical differences by applying a method for deriving the cutoffs of TyG-BMI and NITGB rather than suggesting fixed values.

### 4.1. Limitations

This study had several limitations. First, the number of patients was too small to derive statistically sufficient significance. Thus, we adjusted the significance level (*P-*value < 0.05 to < 0.1) in the ROC curve and the Cox analyses. Second, because of the retrospective study design, we could not fully control for subclinical confounding factors that could affect not only TyG-BMI and NITGB at diagnosis but also all-cause mortality during follow-up. Moreover, we could not provide the serial data regarding TyG-BMI and NITGB from the time of AAV diagnosis to either the date of death or the last visit. Third, although the prevalence rate of AAV has been known to be rare (29, 30), and access to an independent cohort of AAV patients has not been easy, it would have been helpful to validate the predictive ability of NITGB for all-cause mortality in AAV patients with different ethnicities. Nevertheless, our new index of NITGB could not be fully validated in the external validation cohort dataset due to several hurdles such as the COVID-19 pandemic era. Therefore, our findings from the Korean population cannot be extrapolated to other populations directly, which may be a critical limitation of this study. In the near future, we will conduct another study including AAV patients with diverse ethnicities and believe that that study will validate the results of this study and provide more reliable information on the clinical implications of NITGB in AAV patients.

## 5. Conclusion

In conclusion, we demonstrated the clinical implication of TyG-BMII for predicting all-cause mortality, developed a new index for predicting mortality using TyG-BMI, and demonstrated that NITGB at AAV diagnosis along with TyG-BMI could predict all-cause mortality during follow-up in non-obese AAV patients. In addition, the predictive ability of NITGB seemed greater than that of TyG-BMI for all-cause mortality in non-obese AAV patients. Based on these results, in addition to several known initial predictors of all-cause mortality in AAV patients, we suggest deriving the cutoff value of NITGB at diagnosis in each AAV cohort with racial and geographical characteristics and applying it to newly diagnosed non-obese AAV patients.

## Data availability statement

The original contributions presented in the study are included in the article/Supplementary material, further inquiries can be directed to the corresponding authors.

## Ethics statement

The studies involving human participants were reviewed and approved by the Institutional Review Board (IRB) of Severance Hospital (Seoul, Republic of Korea, IRB No. 4-2020-1071). Written informed consent for participation was not required for this study in accordance with the national legislation and the institutional requirements.

## Author contributions

PP and JP carried out the statistical analysis. PP, JH, and S-WL wrote the first draft of the manuscript. PP, JP, SA, JS, and Y-BP collated data. JH and S-WL are responsible for the conception, funding, design of the study, the guarantors of this work and, as such, had full access to all the data in the study, and take responsibility for the integrity of the data and the accuracy of the data analysis. All authors corrected and approved the revisions and final version of the manuscript.
