# Supplementary material for: New index using triglyceride glucose-body mass index for predicting mortality in patients with antineutrophil cytoplasmic antibody-associated vasculitis
Source: Front Med (Lausanne). 2023 May 19;10:1168016. doi: 10.3389/fmed.2023.1168016 (PMC10237337; doi:10.3389/fmed.2023.1168016)
Supplement: Supplementary file 1 [file Data_Sheet_1.docx]

Supplementary Material

# Supplementary Figures


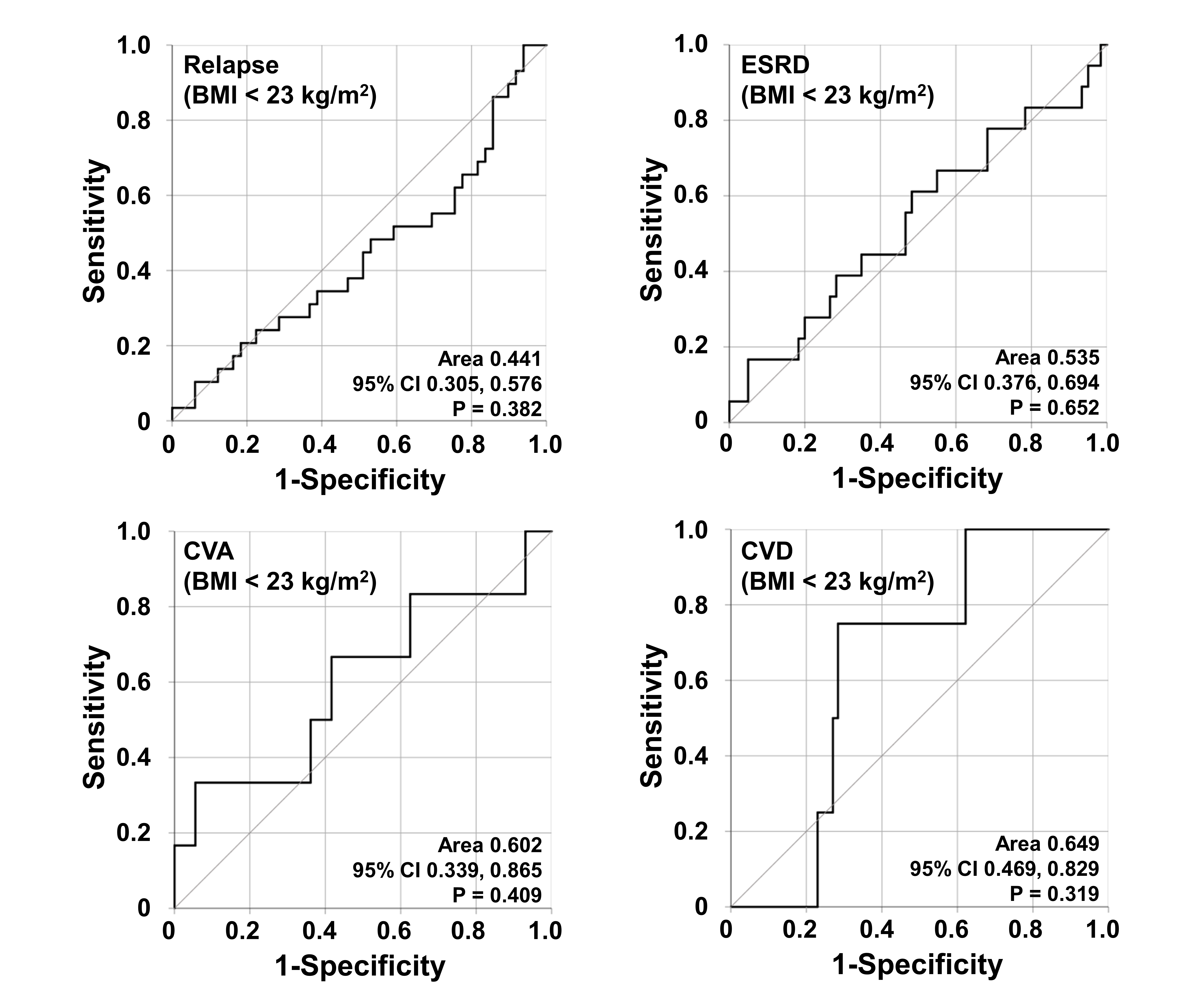


**Supplementary Figure 1.** Comparison of area under the curves of TyG-BMI for poor outcomes other than all-cause mortality in AAV patients with BMI < 23.0 kg/m2

TyG: triglyceride glucose; BMI: body mass index; AAV: ANCA-associated vasculitis; ANCA: antineutrophil cytoplasmic antibody; BMI: body mass index.


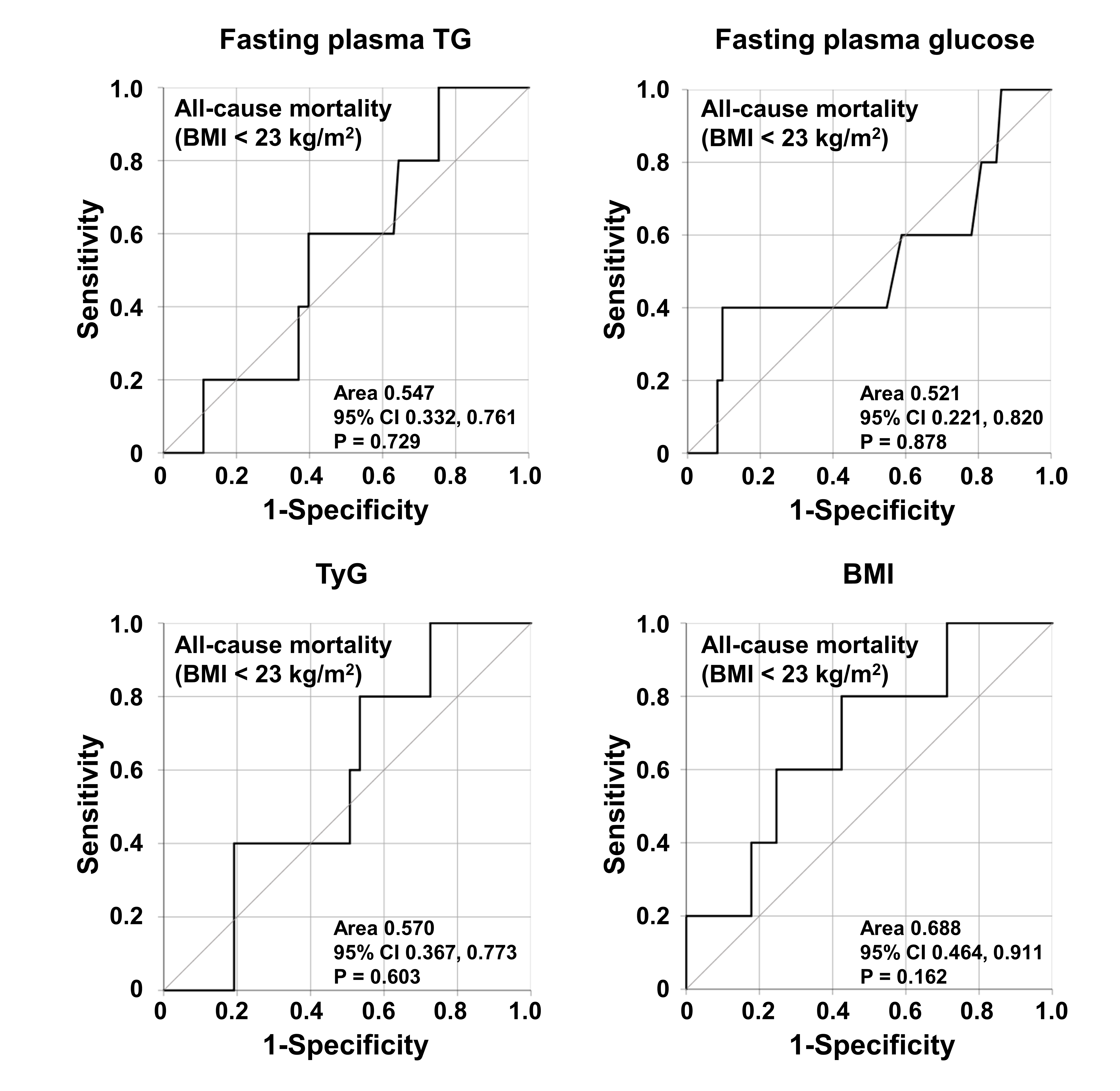


**Supplementary Figure 2**. Comparison of area under the curves of each component composing TyG-BMI for all-cause mortality in AAV patients with BMI < 23.0 kg/m2

TyG: triglyceride glucose; BMI: body mass index; AAV: ANCA-associated vasculitis; ANCA: antineutrophil cytoplasmic antibody; BMI: body mass index.


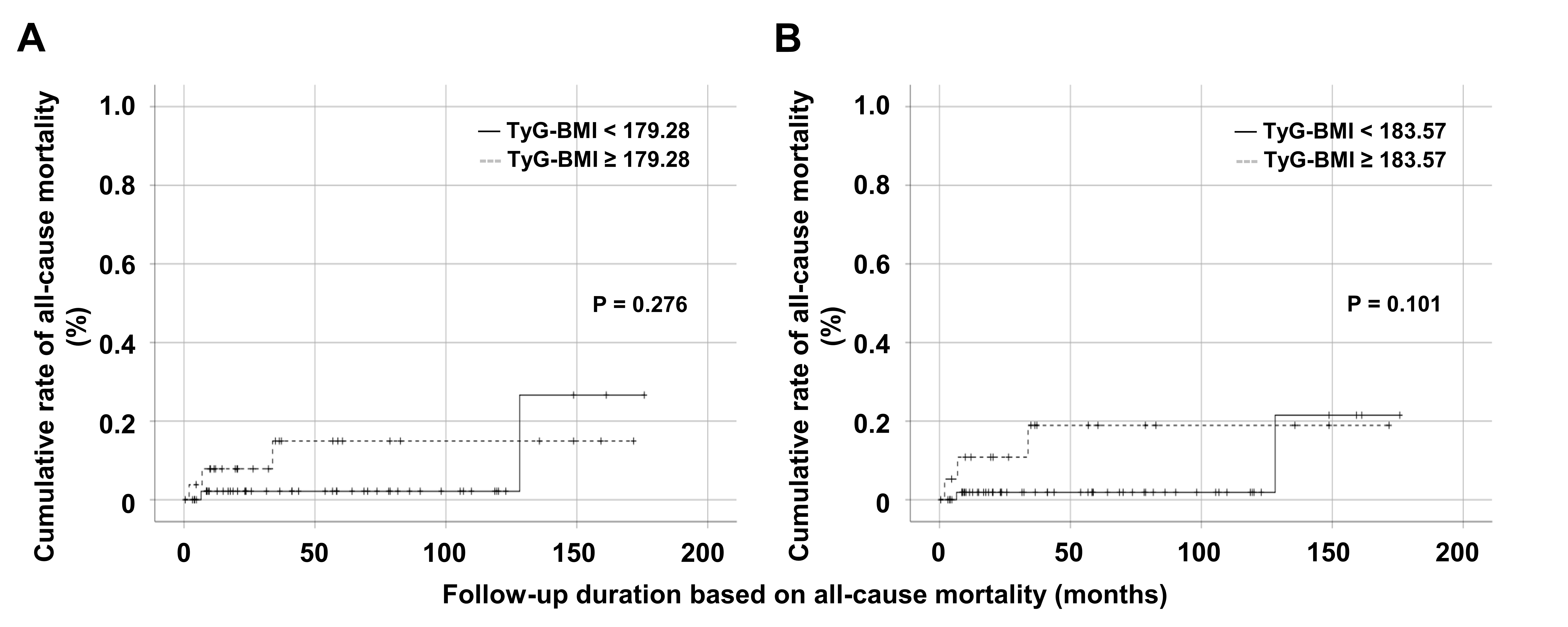


**Supplementary Figure 3**. Comparison of cumulative rates of all-cause mortality based on either the highest tertile or quartile of TyG-BMI in AAV patients with BMI < 23.0 kg/m2

TyG: triglyceride glucose; BMI: body mass index; AAV: ANCA-associated vasculitis; ANCA: antineutrophil cytoplasmic antibody; BMI: body mass index.
